# Kinetics of humoral immune response and severity of infection after three doses of SARS-CoV-2 mRNA vaccine in a large cohort of kidney transplant recipients

**DOI:** 10.1007/s40620-023-01650-8

**Published:** 2023-07-17

**Authors:** Simona Simone, Francesco Pesce, Giulia Fontò, Virginia Pronzo, Paola Pontrelli, Francesca Conserva, Annalisa Schirinzi, Annalisa Casanova, Pasquale Gallo, Michele Rossini, Giuseppe Lucarelli, Marco Spilotros, Maria Rendina, Giovanni Stallone, Francesca Di Serio, Alfredo Di Leo, Silvio Tafuri, Pasquale Ditonno, Loreto Gesualdo

**Affiliations:** 1 Nephrology, Dialysis and Transplantation Unit, Department of Emergency and Organ Transplantation, University of Bari Aldo Moro, Bari, Italy; 2 Clinic Pathology Unit, University Hospital of Bari, Bari, Italy; 3 Section of Gastroenterology, Department of Emergency and Organ Transplantation, University of Bari Aldo Moro, Bari, Italy; 4 Department of Medical and Surgical Sciences, Renal Unit, University of Foggia, Foggia, Italy; 5 Interdisciplinary Department of Medicine, University of Bari Aldo Moro, Bari, Italy; 6 Urology, Andrology and Kidney Transplantation Unit, Department of Emergency and Organ Transplantation, University of Bari Aldo Moro, 70124 Bari, Italy

**Keywords:** SARS-CoV-2, Vaccines, Kidney transplanted patients, Outcome

## Abstract

**Background:**

COVID-19 in kidney transplant recipients is associated with high morbidity and mortality. In this study we aimed to evaluate: (i) the seroconversion rate after BNT162b2 (Pfizer-BioNTech) SARS-CoV-2 vaccine, (ii) factors associated with humoral response, (iii) clinical outcome of COVID-19 in kidney transplanted patients.

**Methods:**

We enrolled a cohort of 743 kidney transplant recipients followed up from March 2020 until April 2022. A subset of 336 patients, who received three-doses of SARS-CoV-2 vaccine, was analyzed in terms of kinetics of humoral immune response and compared to a control group of 94 healthcare workers. Antibody response was tested before vaccination (T_0_), 15 and 90 days after the second dose (T_1_ and T_2_), on the day of the third dose (T_3_) and one month after the third dose (T_4_).

**Results:**

We observed that 66 out of 743 subjects had COVID-19 infection pre-vaccination: 65.2% had severe symptoms, 27.3% were hospitalized (9 deaths), none were asymptomatic. After three doses, 51 patients had COVID-19 infection, 60.8% were asymptomatic, 27.5% reported mild symptoms, 3.9% showed severe symptoms, 7.8% were hospitalized (2 deaths).

In the subset of 336 vaccinated patients, an antibody level > 0.8 U/ml was detected at T_1_, that increased at T_2_ and T_3,_ peaking at T_4_. Independent factors associated with a negative antibody titer at T_4_ were decreasing estimated glomerular filtration rate, time from transplantation, and antimetabolites (all *p* < 0.001) and age (*p* = 0.007).

**Conclusions:**

The kinetics of humoral response after three doses of vaccine in kidney transplant patients is characterized by a late but effective immune response against SARS-CoV-2, reducing morbidity and mortality.

**Graphical abstract:**

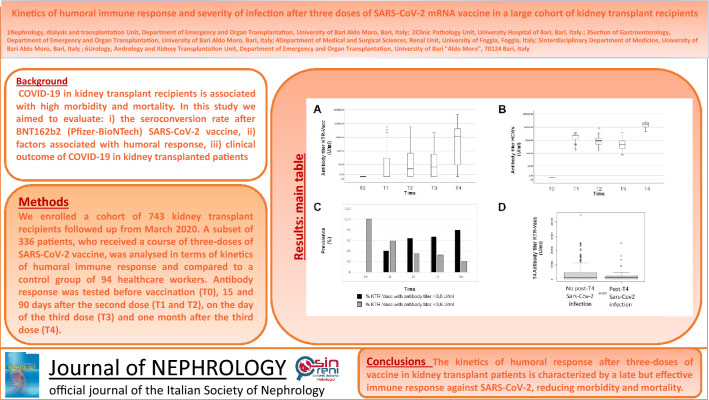

## Introduction

The novel coronavirus epidemic, caused by severe acute respiratory syndrome coronavirus 2 (SARS-CoV-2), originated in Wuhan, a city in China, in December 2019, rapidly spread around the world and was declared a pandemic in March 2020 [1]. Messenger RNA (mRNA) vaccines soon emerged as promising options to reduce the risk of spreading the infection [2].

Immunosuppression and comorbidities account for the higher risk of morbidity and mortality in solid organ transplant recipients with SARS-CoV-2 infection [3]. Several studies showed that a two-dose regimen of mRNA COVID-19 vaccine has low immunogenicity in immunocompromised patients [4, 5] as compared to healthy participants and to those who have chronic stable disease, tested in vaccine trials [6, 7]. In September 2021, the Italian Medicines Agency (AIFA) Technical Scientific Commission (CTS) authorized a third mRNA vaccine administration as an additional primary dose to immunocompromised patients in order to complete the vaccination schedule. A third SARS-CoV-2 vaccine dose is responsible for better humoral response in the solid organ transplant population against all variants, but not against Omicron [8]. Such irregular immune response to vaccination in subjects receiving chronic immunosuppressive therapies was previously observed following other common vaccines including pneumococcal, hepatitis B virus and influenza vaccination [9].

In solid organ transplant recipients, an additional dose of the BNT162b2 vaccine has been associated with an increased response to the SARS-CoV-2 infection [10, 11]. However, a large proportion of patients remain at risk for COVID-19 infection, and the severity of the clinical symptoms in this population has not yet been described. A recent study revealed that a third dose of the BNT162b2 vaccine improves antibody response in kidney transplant recipients [12], although the effectiveness of this approach on clinical outcomes such as death and hospitalization remains unknown. Callaghan et al., conducted a national registry study which showed a lower rate of death after COVID-19 infection among transplant recipients vaccinated with the Oxford-AstraZeneca vaccine than among those vaccinated with BNT162b2 Pfizer [13].

Both kidney transplant recipients and other fragile subjects, such as dialyzed patients, are at high risk of developing severe disease after SARS-CoV-2 infection, and in fact, the ERACODA study demonstrated a higher 28-day case-fatality rate in this population [14].

Thus, in our study, we assessed the intensity and kinetics of humoral response after the administration of the BNT162b2 vaccine, both in kidney transplant recipients and matched healthy controls, and investigated the baseline clinical factors associated with vaccine response. Moreover, we evaluated the impact on clinical outcome, including death, of COVID-19 infection in kidney transplant recipients (KTRs) along with the different doses of vaccination.

## Methods

### Study population

We carried out a longitudinal study involving a single-center cohort of 743 KTRs routinely followed up from March 2020 until April 2022 in the outpatient clinic of the Nephrology, Dialysis and Transplantation Unit, Department of Emergency and Organ Transplantation, University of Bari “Aldo Moro” and a control group of 94 healthcare workers (HCWs) older than 18 years.

Among the enrolled patients, 439 KTRs were administered the COVID-19 vaccination protocol starting in March 2021, receiving three vaccine doses (KTR-VACC). Among these patients we collected biological samples during the indicated time periods (before vaccination (T0), 15 and 90 days after the second dose (T1 and T2), on the day of the third dose (T3) and one month after the third dose (T4)) to evaluate the kinetics of the antibody response. All of these patients were followed up until April 2022 in order to report COVID-19 infections and clinical course.

All KTR-VACCs who provided biological samples signed informed consent forms before enrollment; study procedures conformed to the ethical guidelines of the Declaration of Helsinki and were approved by the Local Ethics Committee (study No. 6845, prot. No. 0050551).

Exclusion criteria were: (i) history of previous positive nasal-swab on a polymerase chain reaction (PCR) assay; (ii) recent transplantation (< 1 year); (iii) chemotherapy treatment within the last year; (iv) changes in immunosuppressive therapy during the post-transplant for rejection events or infections (within 6 months prior to vaccination). Data related to age, gender, ethnicity, and body mass index (BMI), prior history of COVID-19 infection, transplant type and date, medications and comorbid conditions were collected from the patient’s medical records. Cardiovascular events were defined as a history of hypertensive heart disease, angina pectoris, myocardial infarction, and/or stroke. Creatinine levels, estimated glomerular filtration rate ([eGFR], calculated by Chronic Kidney Disease Epidemiology Collaboration [CKD-EPI] equation) and proteinuria were evaluated before and after vaccination. We also recorded data related to COVID-19 infection, including symptoms, classified as mild (cough, cold) or severe (fever, myalgia, asthenia), hospitalization and death.

As a safety measure, a panel reactive antibody test was performed at baseline and after vaccination. Twenty-two patients in the KTR-VACC cohort were excluded because they were classified as SARS-CoV-2 pre-immunized according to the antibody baseline status before receiving the vaccine (T_0_); five patients were excluded because they tested positive for SARS-CoV-2 PCR after the second dose of vaccine. Furthermore, we excluded four patients who died before the administration of the third dose and who showed irregularities in vaccine administration (*n* = 77). In total, 336 KTR-VACCs and the entire HCW control cohort were eligible and included in the analyses.

The COVID-19 vaccination campaign in Italy started in December 2020, and all patients included in the present study were initially vaccinated with two doses of BNT162b2 (Pfizer‐ BioNTech), given at least 21 days apart. Then, according to the recommendations of the AIFA Technical Scientific Commission, an additional vaccine dose (third dose) was administered at least 28 days after the second dose in KTR-VACCs in order to complete the primary vaccination course, while HCWs received the third dose at least 5 months after completing an mRNA vaccine (Pfizer-BioNTech or Moderna) primary series.

Although variant analysis was not performed in our study, these KTRs were infected between March 2021 and April 2022 during which Delta and Omicron were the most prevalent variants in Italy [33].

### Sample collection

Antibody response against the spike protein was tested on blood samples collected before the administration of the first dose of vaccine (T_0_), at 14 and 90 days after the second dose (T_1_ and T_2_ respectively), on the day of the third dose (T_3_, i.e., 6 months after the second dose) and one month after the administration of the third dose (T_4_). Blood samples were collected in BD vacutainer SST II Advance serum collection tubes. Tubes were then centrifuged for 10 min at 3000*g* within 2 h from collection and the resulting serum fraction was transferred into a clean Eppendorf tube and stored at -80 °C until further use.

### Assessment of SARS-CoV-2 Binding Antibody Response and Infection

Samples were tested using the commercially available Elecsys® Anti*-*SARS*-*CoV*-*2* S* assay (Roche Diagnostics International Ltd, Rotkreuz, Switzerland). The Elecsys® Anti-SARS-CoV-2 S assay is a quantitative ECLIA that detects high-affinity antibodies to the SARS-CoV-2 S protein Receptor Binding Domain and has a low risk of detecting weakly cross-reactive and unspecific antibodies. Results are automatically reported as the analyte concentration of each sample in U/ml, with < 0.80 U/ml interpreted as negative for anti-SARS-CoV-2 S antibodies and ≥ 0.80 U/ml interpreted as positive for anti-SARS-CoV-2 S antibodies.

The assay was performed on specimens collected from all participants at different time points as reported above (from T_0_ to T_4_). SARS-CoV-2 infection was defined by a positive result of SARS-CoV-2 PCR on nasal swabs.

### Statistical analysis

Statistical analysis was performed using IBM SPSS statistics for Windows, version 25. Categorical variables are reported as frequencies and percentages, and continuous variables are reported as median and interquartile range (IQR).

The proportion of patients who developed a positive antibody response was assessed by odds ratio (OR) and their 95% confidence intervals (95% CI) by means of univariate logistic regression models taking into account the following variables: age, sex, hypertension, diabetes, cardiovascular disease, time since transplantation, type of donor, eGFR (CKD-EPI), baseline immunosuppression. In order to establish independent factors predicting lack of response to the vaccine, variables that were significantly associated (*p* ≤ 0.05) with the outcome were finally entered into a multivariable logistic model.

## Results

### Characteristics of the study cohorts

Clinical and biological characteristics of the KTR and HCW control groups are detailed in Table 1.

**Table 1:** Baseline characteristics of patients enrolled in the study

Characteristics	KTR (*n* = 743)	Control group (*n* = 94)
Age, yrs	58 [50–68]	53.5 [44–60]
Males, *n* (%)	471 (63.4%)	27 (28.7%)
BMI, kg/m^2^	25.3 [23.0–27.7]	
Re-transplants, *n* (%)	59 (8%)	
Donor type		
DBD	652 (87.8%)	
Living	91 (12.2%)	
Median time since transplantation, yrs	10 [5–16]	
Median eGFR (ml/min/1.73m^2^)	54.1 [38.8–73.6]	
Primary kidney disease, *n* (%)		
Glomerular	198 (26.7%)	
Vascular	20 (2.7%)	
Polycystic kidney disease	87 (11.6%)	
Diabetes	225 (30.3%)	
Cakut	67 (8.9%)	
Kidney stone	11 (1.5%)	
Other	135 (18.3%)	
Maintenance immunosuppressive therapy, *n* (%)		
Calcineurin inhibitors	680 (91.6%)	
Antimetabolite^a^	563 (75.9%)	
Steroids	660 (88.9%)	
mTORi	224 (15.1%)	
Comorbidities, *n* (%)		
Hypertension	645 (86.9%)	
T2D	88 (11.9%)	
CVD	75 (10.1%)	

*DBD* Donation after Brain Death, *eGFR* estimated Glomerular Filtration Rate (Chronic Kidney Disease Epidemiology Collaboration (CKD-EPI) equation), *CAKUT* Congenital Anomalies of the Kidney, *mTORi* Mammalian target of rapamycin inhibitors, *T2D* type 2 diabetes, *CVD* Cardiovascular disease

^a^Includes mycophenolate mofetil, mycophenolic acid, or azathioprine

Among KTRs, median age was 58 [50–68] years, with a predominance of male sex (63.4%), whereas among HCWs, median age was 53.5 [44–60] years, and 71.3% were women. Median time between transplantation and vaccination was 10 [IQR 5–17] years. At baseline, median eGFR was 54.1 [38.8–73.6] ml/min/1.73m^2^ (Table 1). The standard immunosuppressive regimen consisted of glucocorticoids (88.9% of patients), calcineurin inhibitors (91.6% of patients), antimetabolites (75.9% of patients) and mammalian target of rapamycin (mTOR) inhibitors (15.1% of patients). The antimetabolites used were mycophenolate mofetil or mycophenolic acid (94.7%) and azathioprine (5.3%). The third dose was administered 172 ± 4 days after the second dose in KTR-VACCs and 164 ± 4 days after the second dose in HCWs.

### Kinetics of humoral response in transplant patients and healthy controls

**Fig. 1:**
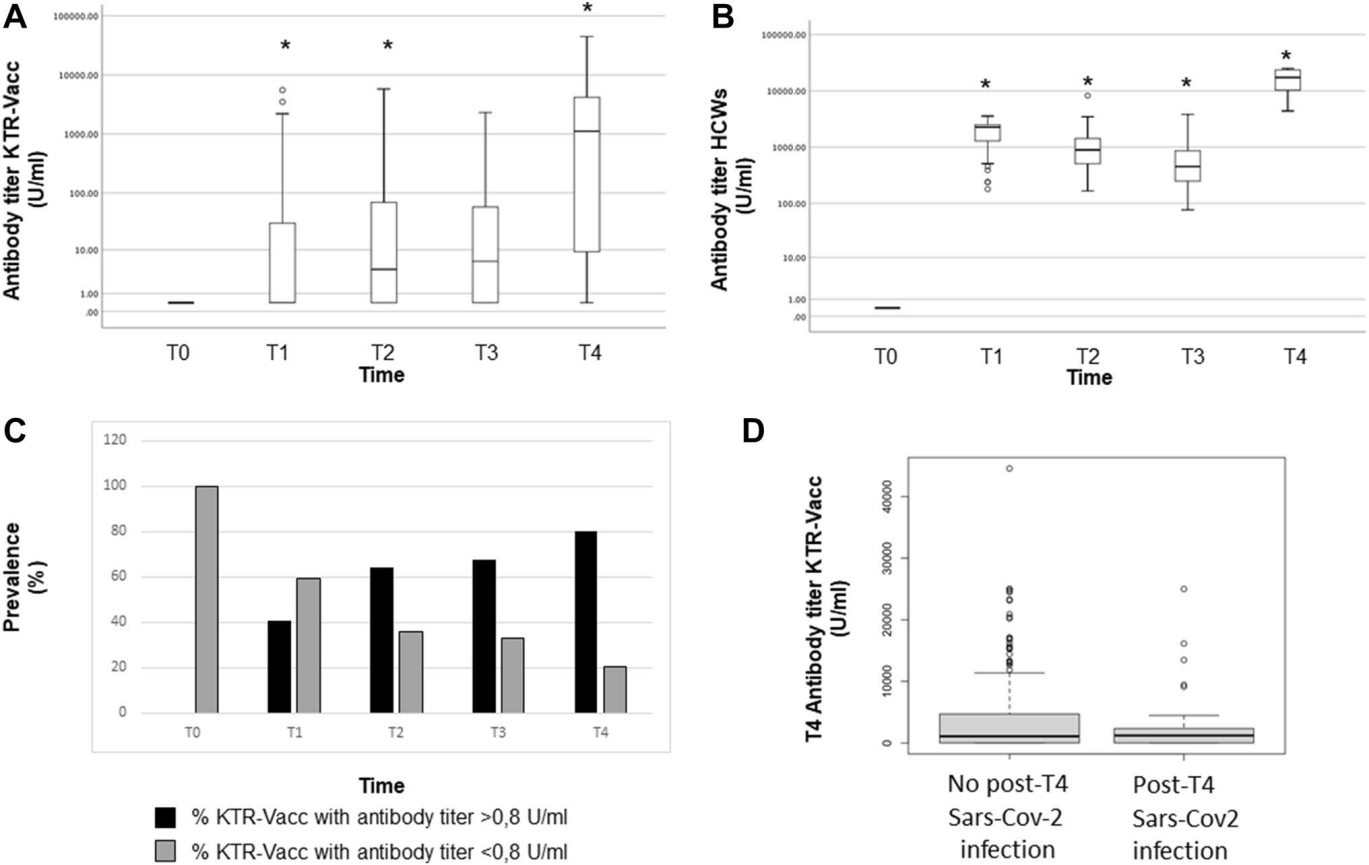
Comparison of antibody titers against SARS-CoV-2 Receptor Binding Domain between KTRs (**A**) and control group (**B**). Course of anti-SARS-CoV-2 S antibody titers for each patient at baseline (T0), at 14 and 90 days after the second dose (T1 and T2, respectively) and the same day and after 1 month subsequent to the third dose (T3 and T4, respectively). Cut-off for positive test was defined according to the manufacturer’s instructions as a titer ≥ 0.8 U/ml. *p < 0.001 vs previous time. **C** Prevalence (%) of responders /non responders to BNT162b2 vaccine among KTRs at different time points (T0, T1, T2, T3 and T4). **D** anti-SARS-CoV-2 S antibody titers at T4 in KTRs who developed COVID-19 infection, and those who did not. *p* = n.s

Both cohorts (KTR-VACCs and HCWs) included only participants with undetectable antibodies against the receptor-binding domain of the SARS-CoV-2 spike protein before vaccination. In KTR-VACCs, an antibody level was detected starting at T_1_ (14 days after the second dose), which progressively increased at T_2_ (90 days after the second dose) and T_3_ (the day of the third dose, 6 months after the second dose), with a peak at T_4_ (one month after the third dose) (Fig. 1A). On the contrary, in HCWs we observed a rapid antibody response after the second dose and a peak after 14 days post injection (median antibody titer at T_1_: 2252 U/ml [1243–2500]) with a significant decrease at T_2_ (90 days post injection: 874 U/mL [504.50–1431.75]) and T_3_ (before the third vaccine dose: 447 U/ml [248.75–858.25]) (Fig. 1B). One month after the administration of the third dose, T_4_, the antibody level in HCWs exponentially increased, reaching the median titer of 16,673 U/ml [10,065.50–23,459.25]. The median antibody titer in the HCW group was significantly higher than that observed in KTR-VACCs at each time point (*p* < 0.001) (Fig. 1A, B). No serious adverse events were reported in either group after each vaccination dose.

A total of 40.8% KTR-VACC patients (137/336) responded to the vaccine at T_1_, with an antibody titer > 0.8 U/ml (median antibody level: 0.4 U/mL [IQR, 040–30.0]). The proportion of responders increased to 64.3% (216/336) at T_2_ (median antibody level: 4.15 U/ml [IQR, 0.40–68.8]) and to 67.3% (226/336) at T_3_ (median antibody level: 6 U/ml [IQR 0.40–57.3]). One month after the third dose, a positive anti-receptor binding domain (RBD) antibody titer was detected in 268 of 336 patients (79.8%) (median antibody titer: 1115 U/ml [IQR, 9.2–4176.5]) (Fig. 1C).

We then analyzed factors associated with an antibody titer < 0.8 U/ml at T_4_ in KTR-VACCs. There was no significant association with type 2 diabetes, hypertension, cardiovascular disease, age or donor type. A multivariate logistic regression analysis confirmed that the independent factors associated with a persistent antibody titer < 0.8 U/ml at T_4_ were: decreasing eGFR (OR [95% CI], 1.051 [1.031–1.070], *p* =  < 0.001), increasing age (OR [95% CI], 0.963 [0.936–0.990], *p* = 0.007), decreasing time from transplantation (years) (OR [95% CI], 1.116 [1.056–1.180], *p* =  < 0.001), therapy with antimetabolites (OR [95% CI], 0.065 [0.016–0.267], *p* =  < 0.001) (Table 2).

**Table 2:** Univariable and multivariable analysis on factors associated with antibody titer < 0.8 U/ml at T4 in KTR-VACCs

	Univariable OR (95% IC)	*p* value	Multivariable OR (95% IC)	*p* value
Age	0.971 (0.949–0.993)	0.009	0.963 (0.936–0.990)	0.007
Sex (female)	1.865 (1.088–3.196)	0.02	0.557 (0.293–1.059)	0.074
Years post-transplant	1.057 (1.016–1.100)	0.007	1.116 (1.056–1.180)	< 0.001
eGFR (ml/min/1.73m^2^)	1.036 (1.021–1.052)	< 0.001	1.051 (1.031–1.070)	< 0.001
Maintenance immunosuppressive therapy				
Antimetabolite maintenance therapy	0.114 (0.035–0.375)	< 0.001	0.065 (0.016–0.267)	< 0.001
mTOR inhibitor maintenance therapy	4.599 (1.385–15.270)	0.01	1.252 (0.288–5.445)	0.764

### Clinical outcomes and severity of symptoms

Among the 743 KTRs, 66 patients tested positive for COVID-19 infection before starting the vaccination program: the large majority (65.2%) had severe symptoms, 27.3% underwent hospitalization (9 patients died), 7.5% showed mild symptoms, none were asymptomatic. Six out of 336 patients had COVID-19 infection after two doses of vaccination. None were asymptomatic and none died following the infection; 2 patients (33.3%) had mild symptoms, 1 patient (16.7%) had severe symptoms, 3 patients (50%) were hospitalized. Interestingly, among the 51 patients who had COVID-19 infection after three doses of vaccination, 60.8% (31 patients) were asymptomatic, thus suggesting the protective role of the three-dose vaccination protocol regardless of the COVID-19 variant. In this group, 14 patients (27.5%) reported mild symptoms, only 2 patients (3.9%) showed severe symptoms, 4 patients (7.8%) were hospitalized and among them 2 patients died. (Table 3). Although there was a trend, we observed that KTR patients who had COVID-19 infection after three doses of vaccination did not show a statistically significant lower humoral response when compared to KTR patients who had no infection (Fig. 1D).

**Table 3:** Severity of COVID-19 infection in the entire KTR cohort

	Pre-vaccine infections (*n* = 66 pts)	Infections after 2 doses (*n* = 6 pts)	Infections after 3 doses (*n* = 51 pts)
Asymptomatic	0%	0%	60.8% (31 pts)
Mild symptoms	7.5% (5 pts)	33.3% (2 pts)	27.5% (14 pts)
Severe symptoms	65.2% (43 pts)	16.7% (1 pt)	3.9% (2 pts)
Hospitalization/death	27.3% (18/9 pts)	50% (3/0 pts)	7.8% (4/2 pts)

## Discussion

The safety and efficacy of three doses of mRNA SARS-CoV-2 vaccines in transplant recipients is still debated [5, 10, 11, 15–17]. Several studies reported that response to vaccination against SARS-CoV-2, like other vaccines, is strongly impaired in immunocompromised patients, such as hemodialyzed subjects, solid organ transplant recipients, HIV infected patients [18, 19], patients with cancer or hematological malignancy undergoing anticancer chemotherapy [20], and those with autoimmune inflammatory rheumatic disease [21].

Available data on antibody response to COVID-19 vaccine in patients receiving maintenance dialysis confirmed a lower and suboptimal humoral response following a 2-dose vaccine regimen than healthy controls [22] and a substantial increase in antibody levels after the additional dose [23, 24]. This evidence led several countries including Italy, to recommend an additional dose of mRNA COVID-19 vaccine in different populations of immunocompromised subjects.

The aim of our study is to compare the kinetics of antibody response, after a three-dose regimen of Pfizer BNT162b2 Comirnaty vaccination in KTR-VACCs, with the response in healthy controls at different time points (3, 6 months after the second dose and 1 month after the third dose). We observed that humoral response was qualitatively and quantitatively different between the two groups, since we found lower median antibody levels after anti–COVID-19 vaccination in KTR-VACCs compared to healthy controls at each time point. These results confirmed the lower humoral response of KTR patients compared to healthy subjects. To the best of our knowledge there are no studies comparing the kinetics of humoral response at different time points in these groups. As described elsewhere [25], we observed that healthy individuals developed a positive antibody response starting from 14 days (T_1_) after the second dose, which dropped progressively at 90 days and 6 months after the second dose, and then finally increased 30 days after the booster dose (Fig. 1B). In contrast, a more granular analysis of the kinetics of humoral response revealed for the first time that the antibody response to SARS-CoV-2 vaccination among KTR-VACCs was blunted and delayed. In KTRs the antibody titer showed a progressive increase from T_1_ to T_4_ without any reduction (Fig. 1A). These data support the hypothesis that in KTRs the administration of a third dose of the BNT162b2 vaccine is needed to strengthen the immunogenicity of the vaccine. In healthy controls however, a third dose restored the humoral response which significantly declined before the planned administration.

It must be noted that in vaccinated KTRs receiving three doses, those who tested positive showed a slightly lower level of antibody titer (Fig. 1D).

Interestingly, we also described the real clinical effectiveness of a three-dose regimen of COVID-19 vaccination in a large cohort of kidney transplant recipients. We analyzed, for the first time, the correlation between clinical outcomes and vaccination status (Table 3). We observed that the large majority of patients who tested positive for SARS-CoV-2 infection before the administration of the COVID-19 vaccine had severe symptoms (65.2%) and were hospitalized; we did not observe any asymptomatic cases. Conversely, after three doses of vaccination, only 3.9% of patients had severe illness and 60.8% of patients were asymptomatic. This evidence supports the efficacy of the three-dose regimen in KTRs for dealing with the severity of COVID-19 infection. Aslam et al. demonstrated that two doses of COVID-19 vaccination in solid organ transplant recipients were able to induce an 80% reduction in the incidence of symptomatic COVID-19 versus unvaccinated solid organ transplant recipients during the same time [26]. Thus, the increased humoral response after a third dose of mRNA vaccine in KTR-VACCs and the increased clinical effectiveness observed in our population as well as in other studies [10, 15, 16], support the extensive use of the third vaccine dose policy in fragile patients, ensuring sustained and long-lasting protection against severe SARS-CoV-2 disease in the community. The third dose of SARS-CoV-2 mRNA vaccination in KTRs was recently described as being associated with an increased antiviral antibody response against different variants of SARS-CoV-2. However, the neutralizing responses to the Omicron variant overall remained markedly diminished [27]. Omicron variants became endemic in Italy starting from January 2022. Interestingly, despite this evidence, in our patient cohort we observed a marked reduction in the severity of clinical manifestations (Table 3) in those patients who had COVID-19 infection after the third dose (observation period: October 2021–April 2022) compared to the severity of the disease before vaccination.

Our data also showed that vaccine response in KTR-VACCs was strongly influenced by increased age, decreased renal function, antimetabolite maintenance therapy and time from transplantation. The importance of age in mRNA-vaccine response was also described by Grupper et al., who showed the influence of age on mRNA-vaccine response in dialysis patients regardless of chronic medical conditions [5] and Campise et al., who reported older age and dyspnea as variables associated with an increased risk of hospitalization [28]. Moreover, persistent but declining anti-SARS-CoV-2 humoral immunity six months after the three-dose vaccination schedule with BNT162b2 in healthy individuals was more pronounced among older people [29]. Impaired renal function impacts negatively on vaccine response mainly due to inflammaging which occurs through different mechanisms [30]. KTR-VACCs under immunosuppressive agents generally have lower rates of seroconversion, lower antibody titer, and a less sustained response after immunization compared to immunocompetent subjects [31]. We previously demonstrated that mTOR-inhibitors can have a potentially beneficial effect as modulators of immune response to COVID-19 vaccine in KTRs [32].

Our study has a few limitations: (a) data on symptoms and hospitalization in the control group were not available, therefore we could not perform a direct comparison with KTRs; (b) the specific SARS-CoV-2 variant was not tested, however given the study period we can assume that the entire cohort was infected with Delta and Omicron variants; (c) we did not assess neutralizing antibody activity and cellular response that might further strengthen our results.

In conclusion, we evaluated the post-vaccine humoral response in KTRs compared to healthy individuals, and assessed the severity of infection according to the vaccination protocol timeline. Our findings support the real-world effectiveness of a three-dose regimen of COVID-19 vaccine in increasing both the immune response and the prevalence of seroconversion rate, and reducing the morbidity and mortality of SARS-CoV-2 infection despite variants.

Future prospective studies are needed to define the long-term effectiveness and immunogenicity of SARS-CoV-2 vaccines in KTRs and to evaluate the importance of subsequent doses of vaccine in this population.

## Data Availability

The data employed for this study are available upon reasonable request.

## Declarations

### Human and animals rights

The study including human participants has been performed in accordance with the ethical standards of the Declaration of Helsinki and its later amendments.
